# Genome-Wide Analysis of Cellulose Synthase Superfamily and Roles of *GmCESA1* in Regulating Drought Tolerance and Growth of Soybean

**DOI:** 10.3390/plants15010034

**Published:** 2025-12-22

**Authors:** Chunhua Wu, Jie Chen, Jiazhou He, Xiujie Zhang, Shanhui Zheng, Yongpeng Pan, Ting Jin, Yan Li

**Affiliations:** State Key Laboratory of Crop Genetics & Germplasm Enhancement and Utilization, Zhongshan Biological Breeding Laboratory, National Center for Soybean Improvement, National Innovation Platform for Soybean Breeding and Industry-Education Integration, Key Laboratory for Biology and Genetic Improvement of Soybean (General, Ministry of Agriculture), Jiangsu Collaborative Innovation Center for Modern Crop Production, Sanya Institute, Nanjing Agricultural University, Nanjing 210095, China; 2018201048@njau.edu.cn (C.W.);

**Keywords:** soybean, cellulose synthase, *GmCESA1*, drought, plant growth

## Abstract

The cellulose synthase (CS) superfamily, comprising the cellulose synthase (CESA) and cellulose synthase-like (CSL) families, plays crucial roles in plant response to abiotic stresses, growth and development. However, there are few reports on the biological functions of CSs in soybean. In this study, 80 soybean CS members were identified and classified into seven subfamilies. Collinearity analyses revealed that the segmental duplication is likely the primary driver for the expansion of CS superfamily in soybean. The abundant stress-responsive and growth-related *cis*-acting elements in the promoter regions of soybean *CS* genes suggest their potential functions. Notably, *GmCESA1* exhibited significantly higher expression levels in drought-tolerant soybean under drought stress. Soybean plants with lower *GmCESA1* expression via virus-induced gene silencing (VIGS-*GmCESA1*) were less drought-tolerant than the control plants (VIGS-EV), showing reduced relative water content and dry weight than VIGS-EV under drought stress. Furthermore, VIGS-*GmCESA1* soybean plants displayed reduced plant height under both well-watered and drought-stressed conditions. Our findings highlight that *GmCESA1* has pleiotropic functions in regulating both drought tolerance and growth in soybean, contributing to our knowledge on CS and providing a valuable gene to breed drought-tolerant soybean in the future.

## 1. Introduction

Soybean (*Glycine max* (L.) Merr.) is a leguminous crop that serves as a major source of vegetable oil, protein, feed, and industrial raw materials [1]. However, drought adversely affects soybean throughout all growth and development stages [2] and can even lead to yield losses of up to 40% [3]. Therefore, identification of drought-tolerant genes and development of drought-tolerant soybean varieties are critical to ensure sustainable soybean production.

Plants subjected to drought stress typically exhibit leaf wilting and dehydration. Relative water content (RWC) serves as a key indicator of leaf water status, with higher RWC values generally corresponding to enhanced drought tolerance [4]. The abscisic acid (ABA) signaling pathway plays a central role in plant response to drought stress [5]. Drought induces ABA accumulation [6], which activates downstream signaling cascades [7]. Specifically, ABA binds to receptor complexes composed of PYR/PYL/RCAR (Pyrabactin resistance 1/PYR1-like/Regulatory component of ABA receptors) and PP2C (Clade A type 2C protein phosphatases), thereby relieving PP2C-mediated inhibition of SnRK2s (SNF1-related protein kinase 2). The activated SnRK2s phosphorylate transcription factors such as MYC, MYB, and NAC, leading to the expression of ABA-responsive genes and associated molecular mechanisms [8,9]. Osmotic stress often accompanies drought conditions [10]. In *Arabidopsis thaliana*, for example, ten SnRK2s—excluding SnRK2.9—have been shown to respond to osmotic stress [11]. Additionally, drought triggers the accumulation of reactive oxygen species (ROS), which function as important signaling molecules that promote stomatal closure [12]. However, excessive ROS levels can elevate malondialdehyde content and increase membrane permeability [13,14], while also promoting cross-linking between phenolic compounds and cell-wall glycoproteins—a process that contributes to cell wall rigidification [15].

The plant cell wall serves as the primary physical barrier against abiotic stresses and plays a crucial role in stress sensing and signal transduction [16,17,18]. A major component of the plant cell wall is cellulose, which accounts for approximately 20% to 30% of the dry weight of primary cell wall and about 50% of secondary cell wall [19,20,21,22]. The basic structural unit of cellulose is the microfibril, consisting of β-1,4-glucan chains synthesized by cellulose synthase (CESA) [23]. Through hydrophobic cluster analysis, researchers identified D, D, D, and QXXRW motifs of CESA [24], which aided in the discovery of the first plant cellulose synthase genes, *celA1* and *celA2*, in cotton [25]. Subsequently, CESA proteins have been characterized in other prokaryotes and seed plants [26]. The cellulose synthase (CS) superfamily comprises the CESA family and the cellulose synthase-like (CSL) families [27]. In *A. thaliana*, AtCESA1, AtCESA2, AtCESA3, AtCESA5, AtCESA6 and AtCESA9 are considered essential for cellulose synthesis in the primary cell wall [28,29], whereas AtCESA4, AtCESA7 and AtCESA8 are essential for cellulose synthesis in secondary wall [30,31]. Certain CSL subfamilies, such as CSLA, CSLC, CSLD, CSLF, CSLH, and CSLJ, are involved in the synthesis of non-cellulosic polysaccharides [32,33,34,35,36], while the functions of other CSL subfamilies remain unknown.

Accumulating evidence links CS superfamily to abiotic stress tolerance across various plant species. For instance, loss-of-function mutants *cesa1^rsw1-1^* and *cesa6^she1^* were salt-sensitive [37,38]. In contrast, the *lew2* mutant, which carries a mutation in *AtCESA8*, demonstrated improved drought and osmotic tolerance, which was associated with elevated levels of abscisic acid, proline, and soluble sugars compared to wild-type *A. thaliana* [39]. The expression of *CSLA* genes in *Dendrobium officinale* and *Fritillaria cirrhosa* was modulated by PEG, NaCl, and Cd treatments [40,41], suggesting their potential roles in abiotic stress responses. In *Amorphophallus konjac*, the expression level of *AkCSLA11* was influenced by drought-stress (DS), and its overexpression in *A. thaliana* led to increased water loss and reduced survival rate under DS. The proposed mechanism suggests that *AkCSLA11* inhibits the expression of stress-related genes (*AtCOR15A*, *AtRAB18*, *AtSnRK2.6*, *AtP5CS*, *AtPSCS1*, and *AtAREB1*) and alters the sugar components of cell walls [42]. Furthermore, *AtCSLD5* mutant *sos6-1* displayed hypersensitivity to osmotic stress and accumulated elevated ROS levels, indicating that *AtCSLD5* plays a role in regulating osmotic stress tolerance of *A. thaliana* [43].

Despite the documented roles of CS in abiotic stress responses in *A. thaliana* and other species, a systematic analysis of the CS superfamily and its functional relevance to drought tolerance in soybean remains largely unexplored. To address this knowledge gap, we performed a genome-wide analysis of the CS superfamily in soybean. The CS members were identified and subjected to comprehensive bioinformatics analyses. As *AtCESA1* encodes an essential subunit of the primary wall cellulose synthase complex in *A. thaliana* [37,44], we aimed to elucidate the biological function of its soybean homolog *Glyma.06G069600* (*GmCESA1*) in this study. Notably, differential expression of *GmCESA1* was observed between the drought-tolerant soybean PI595843 (PI) and the drought-sensitive soybean Nan Tong Xiao Yuan Dou (NT) under a drought condition. We demonstrated that silencing *GmCESA1* resulted in compromised drought tolerance and reduced plant height in soybean. Furthermore, functional characterization of *AtCESA1* revealed its influence on both drought tolerance and growth in *A. thaliana*. The conserved function of *CESA1* in soybean and *A. thaliana* highlights its potential as a promising candidate gene for breeding sustainable crops.

## 2. Results

### 2.1. Identification and Phylogenetic Analyses of the CS Superfamily in Soybean

To identify members of the CS superfamily in soybean, we employed the Hidden Markov Model (HMM) profile CESA (PF03552) as a query to screen the soybean genome (Wm82.a2.v1). Additionally, BLAST searches were performed in Phytozome V14 using all known *A. thaliana CESA* and *CSL* genes as queries to supplement the candidate gene set. After verification of the “Cellulose_syn” domain using the SMART online tool, a total of 80 non-redundant CS superfamily members were identified (Supplementary Table S1).

The physicochemical properties of these soybean CS proteins exhibit considerable diversity (Supplementary Table S1). They vary in length from 186 amino acids (Glyma.18G098100) to 1336 amino acids (Glyma.05G160000), with molecular weight ranging from 21,074.84 Da to 149,983.70 Da. The isoelectric point spans from 5.64 (Glyma.08G117500) to 9.56 (Glyma.18G098100). Most CS proteins are predicted to lack signal peptide but contain transmembrane domain.

**Figure 1:**
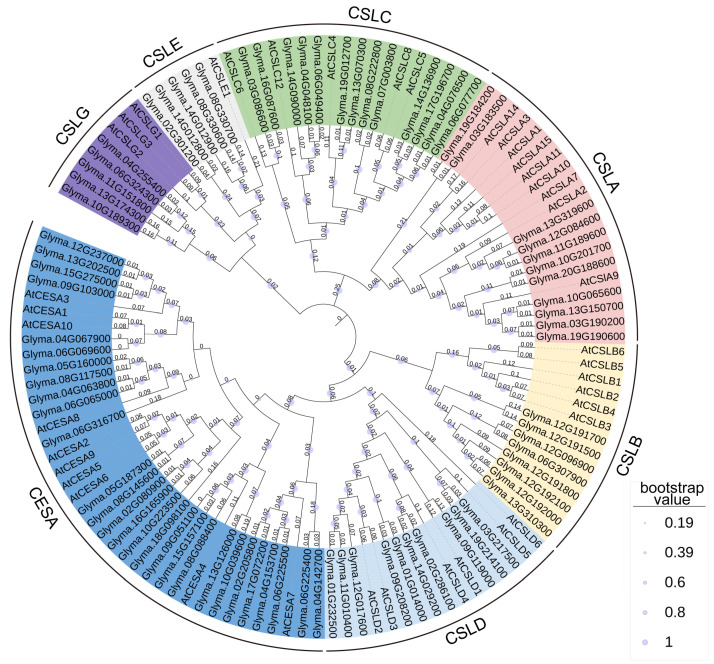
Phylogenetic tree of the CS superfamily in soybean and *Arabidopsis thaliana*. The tree was constructed using the Neighbor-Joining method in MEGA 7.0. Bootstrap values (based on 1000 replicates) are indicated by purple circles on the branches, while numerical labels represent branch lengths. The seven distinct colors correspond to different subfamilies associated with the respective clades.

Phylogenetic analysis classifies these 80 members into seven subfamilies: CESA, CSLA, CSLB, CSLC, CSLD, CSLE, and CSLG (Figure 1). The CESA subfamily is the largest, containing 28 members, followed by CSLC (13 members), CSLA (11), CSLD (10), CSLB (8), CSLE (5), and CSLG (5).

### 2.2. Gene Structures and Conserved Motifs of the Soybean CS Superfamily

The gene structural characteristics of the CS superfamily were analyzed by examining exon–intron organization using the online GSDS2.0 software (Supplementary Figure S1). The number of exons ranges from 3 to 25, while introns vary between 2 and 24. Gene structure is highly conserved within specific subfamilies. For example, all members of the CSLA and CSLB subfamilies contain 9 exons and 8 introns. In contrast, members of CESA, CSLC, CSLD, CSLE, and CSLG subfamilies exhibit considerable diversity in gene structure, with substantial variation in exon and intron numbers within each subfamily.

Conserved motifs within the CS protein sequences were identified using the MEME online tool (Supplementary Figure S2). Motif composition is highly conserved within the CSLA, CSLB, and CSLC subfamilies, suggesting potential functional similarity among members of the same subfamily. On the contrary, CESA, CSLD, CSLE, and CSLG subfamilies display variable motif compositions.

### 2.3. Intraspecific and Interspecific Collinearity of CS Genes

**Figure 2:**
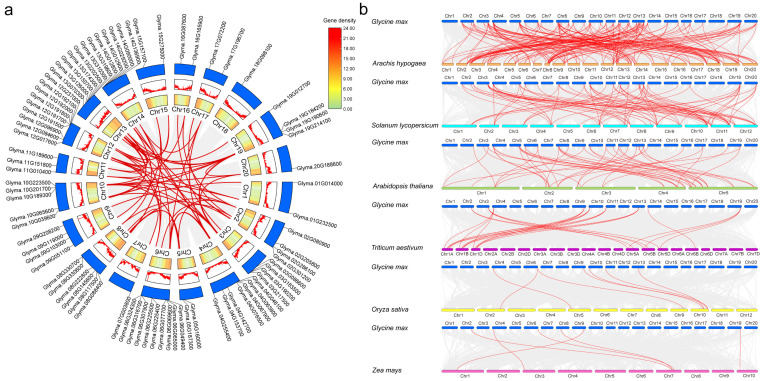
Collinearity analysis of *CS* genes. (**a**) Intraspecific collinearity of *CS* genes within *Glycine max*. (**b**) Interspecific collinearity of *CS* genes between *G. max* and other plant species, including *Arachis hypogaea*, *Solanum lycopersicum*, *Arabidopsis thaliana*, *Triticum aestivum*, *Oryza sativa*, and *Zea mays*. The gray solid lines in the background represent all genome-wide collinear gene pairs within or across species, while red solid lines highlight collinear *CS* gene pairs.

The 80 identified *CS* genes are distributed across all 20 chromosomes of soybean (Figure 2a). Chromosomes 6 and 12 contain the highest number of *CS* genes, with nine members each, while chromosomes 7, 18, and 20 have the lowest, with only one member each. The remaining chromosomes harbor between two and five *CS* genes (Figure 2a, Supplementary Table S1).

In the evolution of gene family, gene duplication serves as a major driver of gene family expansion [45]. Three tandem duplication events were identified: *Glyma.06G225400*/*Glyma.06G225500*, *Glyma.12G191700*/*Glyma.12G191800*, and *Glyma.12G192000*/*Glyma.12G192100* (6 out of 80 genes, 7.50%). Additionally, a total of 63 segmental duplication pairs involving 33 *CS* genes were detected (33/80, 41.25%). Among these, *Glyma.10G065600* exhibits the highest number of segmental duplication events, participating in four duplication pairs (Figure 2a). These results indicate that segmental duplication acts as the predominant mechanism for the expansion of the CS superfamily in soybean.

To evaluate the selection pressure on CS superfamily in soybean, we calculated the Ka/Ks ratios for each gene pair. The Ka values (nonsynonymous substitutions) ranged from 0.003 to 0.401 (mean = 0.086), and Ks values (synonymous substitutions) varied from 0.068 to 2.432 (mean = 0.681). The resulting Ka/Ks ratios for 63 gene pairs ranged from 0.029 to 0.534, with a mean of 0.133, indicating that the evolution of *CS* genes in soybean is likely predominantly driven by purifying selection (Supplementary Table S2).

Additionally, collinearity analyses between soybean and six other species identified 48, 39, 33, 9, 6, and 6 homologous *CS* gene pairs between *Glycine max* and *Arachis hypogaea*, *Solanum lycopersicum*, *Arabidopsis thaliana*, *Triticum aestivum*, *Oryza sativa* and *Zea mays*, respectively (Figure 2b). These results suggest a closer evolutionary relationship between the *CS* genes of soybean and those of *A. hypogaea*.

### 2.4. Cis-Acting Elements in CS Gene Promoters

**Figure 3:**
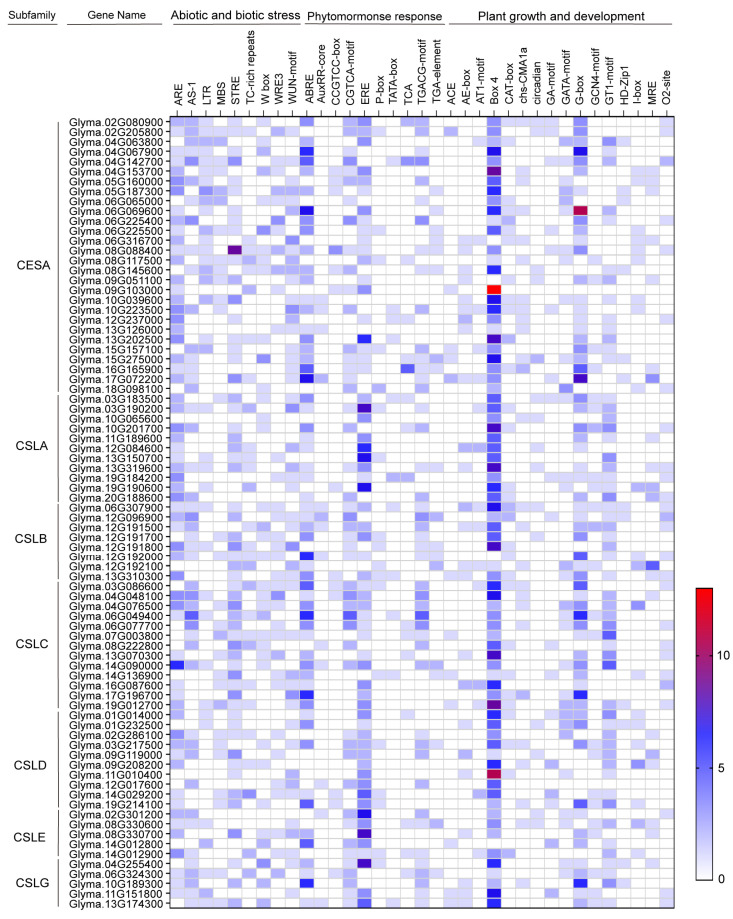
Profile of *cis*-acting elements in the promoter regions of soybean *CS* genes. The color scale represents the numbers of *cis*-acting elements identified.

To explore the potential functions of *CS* genes, we analyzed the *cis*-acting elements within their promoter regions (the 2000 bp upstream of the transcription start site) using the PlantCARE database. The identified *cis*-acting elements are categorized into three groups: abiotic and biotic stress, phytohormone response, plant growth and development (Figure 3). In the abiotic and biotic stress group, *cis*-acting elements such as AS-1 (present in 46 out of 80 *CS* genes), MBS (29/80), STRE (57/80), and W box (35/80) have been associated with drought tolerance in plants [46,47,48,49], suggesting their potential roles in plant response to drought. The phytohormone response group comprised 10 types of *cis*-acting elements, including ABRE, AuxRR-core, CCGTCC-box, CGTCA-motif, ERE, P-box, TATC-box, TCA, TGACG-motif, and TGA-element. Of these, ABRE (associated with abscisic acid response) was the most prevalent (58/80). The growth and development group contained 16 *cis*-acting elements, such as Box-4 and G-box. The abundance and diversity of these *cis*-acting elements suggest that *CS* genes are likely involved in multiple biological processes, including stress response, phytohormone signaling, and growth regulation.

### 2.5. Expression Patterns of CS Genes in Different Soybean Tissues

The expression patterns of *CS* genes were analyzed across eight soybean tissues—pod, leaf, root, nodule, seed, shoot apical meristem (SAM), stem, and flower—using publicly available RNA-Seq data. The soybean *CS* genes display diverse expression profiles (Supplementary Figure S3). Certain genes, such as *Glyma.03G183500*, *Glyma.09G208200*, *Glyma.10G201700*, and *Glyma.13G126000*, exhibit highly tissue-specific expression patterns, with high abundance in a single tissue but little or no expression in others. In contrast, some *CS* genes, including *Glyma.02G080900*, *Glyma.03G190200*, *Glyma.04G067900*, *Glyma.05G187300*, and *Glyma.06G069600*, show high expression levels in multiple tissues. Conversely, several *CS* genes, such as *Glyma.02G286100*, *Glyma.06G307900*, *Glyma.06G316700*, *Glyma.10G039600*, *Glyma.14G029200*, *Glyma.18G098100*, and *Glyma.19G184200*, remain undetectable or have low expression levels in all tissues examined.

### 2.6. GmCESA1 Exhibited Higher Expression Level in the Drought-Tolerant Soybean Accession PI

**Figure 4:**
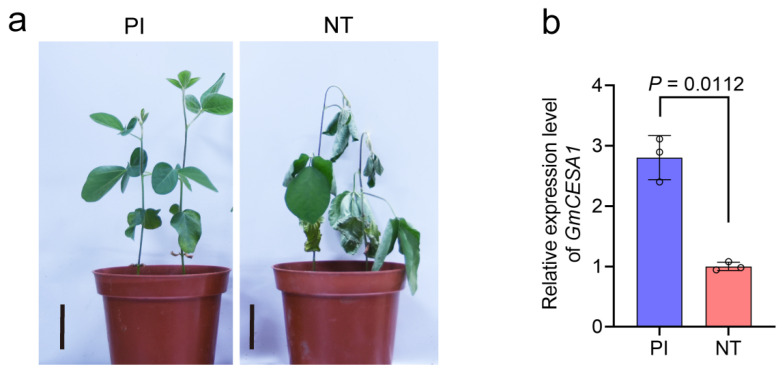
Phenotype and *GmCESA1* expression level in two different soybean accessions under drought stress. (**a**) Shoot phenotypes of PI and NT at 14 days after water was withheld. Bars = 5 cm. (**b**) Relative expression level of *GmCESA1* under drought condition, normalized to NT sample, using *Gm60S* as the reference gene. Data are presented as mean ± SD, *n* = 3 independent biological replicates. Statistical significance was determined using a two-tailed Student’s *t*-test.

Previous studies demonstrate that *AtCESA1* encodes an essential subunit of the primary wall cellulose synthase complex and plays multiple roles in abiotic stress responses in *A. thaliana* [37,44]. To investigate the function of its soybean homolog, *GmCESA1* (*Glyma.06G069600*), we analyzed its expression in a drought-tolerant soybean accession (PI) and a drought-sensitive soybean accession (NT) under DS (Figure 4a). The expression of *GmCESA1* was significantly higher in PI than in NT under DS (Figure 4b), implying a potential role for *GmCESA1* in soybean drought tolerance.

### 2.7. GmCESA1 Modulates Drought Tolerance and Growth of Soybean

**Figure 5:**
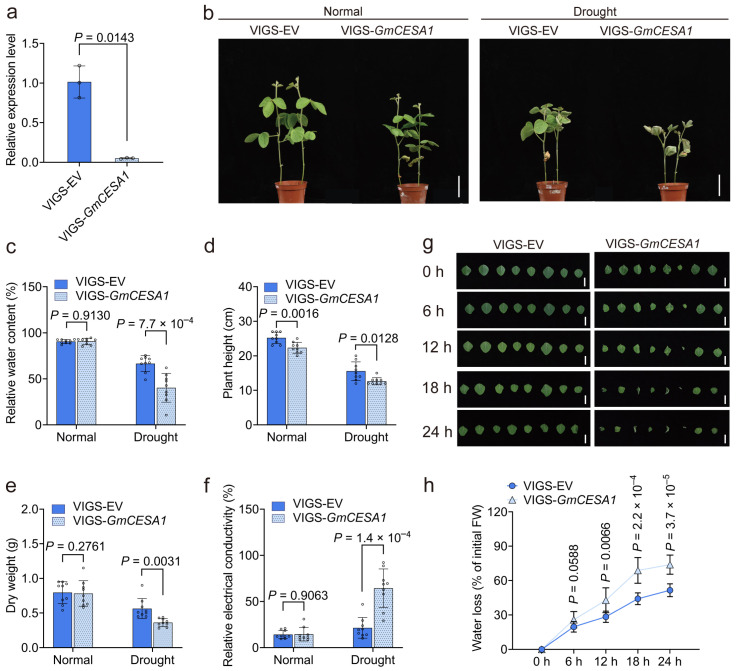
Silencing *GmCESA1* compromises drought tolerance and growth of soybean. (**a**) Relative expression level of *GmCESA1* in soybean plants. Data were normalized to the control plants (VIGS-EV), with *Gm60S* as the reference gene. *n* = 3 independent biological replicates. (**b**), Phenotypes of VIGS-*GmCESA1* and VIGS-EV plants under normal and drought conditions (simulated by 20% PEG6000 treatment for 21 days). Scale bar = 5 cm. (**c**–**f**) Physiological parameters: relative water content (**c**), plant height (**d**), dry weight (**e**), and relative electrical conductivity (**f**) of plants under normal and drought conditions (*n* = 9 plants). (**g**,**h**) Detached leaf assays: images ((**g**), bars = 5 cm) and water loss (% of initial FW) ((**h**), *n* = 8) of detached leaves from VIGS-*GmCESA1* and VIGS-EV plants at different time points. FW, fresh weight. In (**a**,**c**–**f**,**h**) data represents mean ± SD. Statistical significances were determined using two-tailed Student’s *t*-tests.

To investigate the function of *GmCESA1*, we generated *GmCESA1*-silenced plants using the *Bean pod mottle virus* (BPMV)-based virus-induced gene silencing (VIGS) system. RT-qPCR analysis confirmed that the expression level of *GmCESA1* in the VIGS-*GmCESA1* plants was significantly reduced, reaching only 5.05% of that in the empty vector (VIGS-EV) control plants (Figure 5a).

After 10 days of drought treatment, pronounced phenotypic differences were observed between the VIGS-*GmCESA1* and VIGS-EV lines. The *GmCESA1*-silenced plants exhibited severe leaf wilting, dehydration, and chlorosis, whereas the VIGS-EV plants remained relatively turgid and healthy (Figure 5b). Drought tolerance was assessed by measuring relative water content (RWC), plant height, dry weight, and relative electrical conductivity (REC). Under the drought condition, the VIGS-*GmCESA1* plants showed a 26.23% decrease in RWC (Figure 5c), an 18.57% reduction in plant height (Figure 5d), a 35.43% decline in dry weight (Figure 5e), and a 42.90% increase in REC (Figure 5f) compared to the VIGS-EV control. These results demonstrate that silencing *GmCESA1* compromises drought tolerance in soybean.

Leaflets of VIGS-*GmCESA1* plants wilted significantly faster than those of VIGS-EV plants at all observed time points (Figure 5g). Consistent with this phenotype, the detached leaves of VIGS-*GmCESA1* plants exhibited significantly more water loss, which were 6.23%, 14.43%, 24.74%, and 22.35% greater than those of VIGS-EV plants at 6, 12, 18, and 24 h after detachment, respectively (Figure 5h). These results suggest that *GmCESA1* modulates soybean drought tolerance through regulating water loss.

Under normal conditions, no significant differences were observed in RWC, REC, or dry weight between VIGS-*GmCESA1* and VIGS-EV plants. However, the plant height of VIGS-*GmCESA1* was significantly reduced compared to that of VIGS-EV (Figure 5d), indicating that *GmCESA1* also plays a role in the normal growth and development of soybean.

### 2.8. The Arabidopsis cesa1^rsw1-1^ Mutant Is Hypersensitive to Drought and Exhibits Impaired Growth

**Figure 6:**
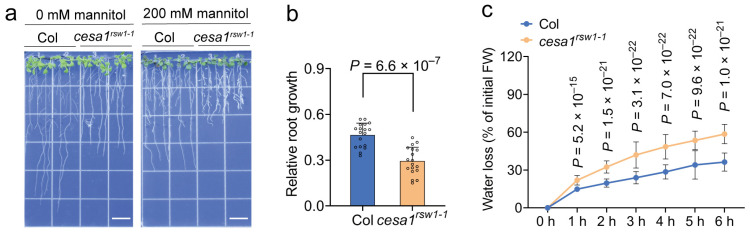
The *cesa1^rsw1-1^* mutant is hypersensitive to drought. (**a**,**b**) Root phenotypes ((**a**), bars = 1 cm) and relative root growth ((**b**), *n* = 18 plants) of Col and *cesa1^rsw1-1^* seedlings grown under control (0 mM mannitol) and simulated drought (200 mM mannitol) conditions for five days. (**c**) Water loss (expressed as percentage of initial fresh weight, FW) of the detached leaves from Col and *cesa1^rsw1-1^* plants over time. Col, *n* = 20 plants; *cesa1^rsw1-1^*, *n* = 22 plants. In (**b**,**c**), data are presented as mean ± SD. Statistical significances were determined using two-tailed Student’s *t*-tests.

The *A. thaliana cesa1^rsw1-1^* mutant, which carries a loss-of-function mutation in *AtCESA1*, showed increased sensitivity to drought stress (simulated by 200 mM mannitol) compared to the wild-type Col (Figure 6a). The relative root growth of *cesa1^rsw1-1^* was significantly lower than that of Col (Figure 6b). In water loss assays using rosette leaves detached from 21-day-old plants, the *cesa1^rsw1-1^* mutant displayed more water loss, which was 7.03%, 12.78%, 16.80%, 18.56%, 20.97%, and 22.26% more than Col at 1, 2, 3, 4, 5, and 6 h after excision, respectively (Figure 6c). This elevated water loss likely underlies the *cesa1^rsw1-1^* mutant’s reduced capacity to retain water under drought conditions.

**Figure 7:**
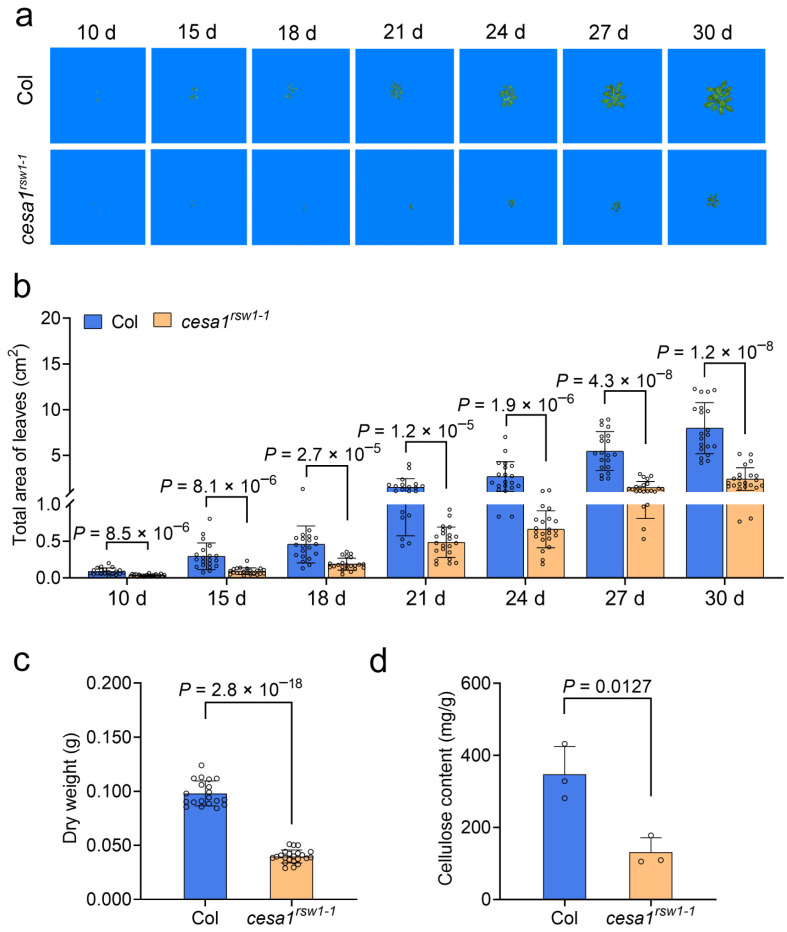
The *cesa1^rsw1-1^* mutant exhibits impaired growth. (**a**) Images of rosette leaves from *Arabidopsis thaliana* plants grown under normal condition for 10 d, 15 d, 18 d, 21 d, 24 d, 27 d and 30 d. (**b**–**d**) Total area (**b**), dry weight (**c**), and cellulose content (**d**) of rosette leaves from Col and *cesa1^rsw1-1^*. In (**b**,**c**), *n* = 20 plants for Col, *n* = 22 plants for *cesa1^rsw1-1^*, in (**d**), *n* = 3 biological replicates for each line. DW, dry weight. In (**b**–**d**), data are presented as mean ± SD. Statistical significances were determined using two-tailed Student’s *t*-tests.

Under normal growth condition, rosette growth of the *cesa1^rsw1-1^* mutant and wild-type Col plants was assessed using the PhenoMate High-throughput Small Plant Phenotype System at 10 d, 15 d, 18 d, 21 d, 24 d, 27 d, and 30 d post-germination. Distinct morphological differences between the mutant and wild-type were evident throughout development (Figure 7a). The total rosette leaf area of *cesa1^rsw1-1^* was 59.10–75.29% smaller than that of Col across all time points (Figure 7b). By day 30, the mutant also exhibited significant reductions in rosette dry weight (by 59.39%; Figure 7c) and cellulose content (by 62.33%; Figure 7d) compared to the wild-type. Collectively, these results indicate that *AtCESA1* is essential for normal growth of *A. thaliana*, likely through its role in cellulose biosynthesis.

## 3. Discussion

Cellulose, a major structural component of the plant cell wall, is synthesized by cellulose synthase [50]. Accumulating evidence suggests that CS members play roles in plant response to abiotic stresses, growth and development [39,51,52]. However, the biological functions of the CS superfamily in soybean remained largely unexplored. In this study, bioinformatic analyses reveal that the promoters of most soybean *CS* genes contain key stress-responsive elements, such as ARE, STRE, and WUN motif (Figure 3), suggesting their potential involvement in abiotic stress adaptation of soybean. In addition, differential expression of *GmCESA1* in a drought-tolerant accession (PI) and a drought-sensitive accession (NT) under drought conditions was observed (Figure 4). These findings implicate the role of *GmCESA1* in soybean response to drought stress. Further experiments showed that *GmCESA1*-silenced soybean plants are less drought-tolerant, supporting the function of *GmCESA1* in regulation of drought tolerance.

Drought stress substantially alters the composition and structure of plant cell wall, which is primarily composed of cellulose, lignin, and pectin [21,22]. In response to drought, the expression of cell wall peroxidase is up-regulated [53]. Elevated peroxidase activity, together with increased ROS levels, may promote the cross-linking of cell wall components, reinforcing cell wall mechanical strength [54]. An increase in pectin content is often observed in plant response to drought [55]. Concurrently, drought stress induces lignin accumulation through the upregulation of genes encoding key enzymes in lignin biosynthesis (such as 4-coumarate: coenzyme A ligase and caffeoyl coenzyme A O-methyltransferase) [56,57]. This enhanced lignin deposition stiffens the cell wall and restricts its extensibility, which helps maintain plant growth under stress conditions [58]. Furthermore, a study in cotton shows that drought increases the content of UDP-glucose for cellulose synthesis [59], while in rice, the COBRA-like protein DROUGHT 1 improves drought tolerance by adjusting cell wall structure through increased cellulose content [60]. A detailed analysis of the cell wall structure of *GmCESA1*-silenced soybean plants would help elucidate the role of *GmCESA1* in mediating drought tolerance.

*AtCESA1* encodes an essential subunit of the primary wall cellulose synthase complex [37,44]. The *cesa1^rsw1^* mutant has been reported to exhibit temperature-sensitive [29] and salt-sensitive [37]. In this study, we found that the *cesa1^rsw1-1^* mutant is less drought-tolerant (Figure 6) and has reduced cellulose content (Figure 7d). Similarly, silencing *GmCESA1* in soybean also significantly compromised drought tolerance, as indicated by decreased RWC, plant height, and dry weight, along with increased REC under the drought condition (Figure 5a–f). Consistently, both *GmCESA1*-silenced soybean plants and the *cesa1^rsw1-1^* mutant exhibited more water loss compared to their controls (Figure 5h and Figure 6c), indicating that *CESA1* regulates drought tolerance through modulating water loss of plants. Given the central role of stomata in transpiration [61,62], future research should elucidate how GmCESA1 modulates water loss, potentially through the regulation of stomatal closure under drought conditions.

CESA subfamily is crucial for normal plant growth. For example, RNAi-mediated suppression of *PtrCESA4*, *PtrCESA7-A/B*, or *PtrCESA8-A/B* resulted in reduced plant height and stunted growth in poplar [63]. Conversely, overexpression of *AtCESA2*, *AtCESA5*, or *AtCESA6* increased plant height and dry weight in *A. thaliana* [52]. Our results align with these reports: *GmCESA1*-silenced soybean plants were significantly shorter than control plants (Figure 5b), and the *cesa1^rsw1-1^* mutant exhibited markedly reduced rosette area and dry weight compared to the wild-type (Figure 7a–c). Together, these results highlight an evolutionarily conserved role for CESA1 in regulating plant growth across species.

## 4. Materials and Methods

### 4.1. Identification and Phylogenetic Analysis of Soybean CS Genes

To identify members of the CS superfamily in soybean, the protein sequence file (Gmax_275_Wm82.a2.v1.protein.fa) was downloaded from Phytozome V14 (https://phytozome-next.jgi.doe.gov/ accessed on 14 May 2024). The Hidden Markov Model (HMM) file for CESA (PF03552) was obtained from the Pfam database (http://pfam.xfam.org accessed on 14 May 2024) and subsequently employed to search the soybean proteome through HMMER3.0 software with a threshold of *E*-values < 1× 10^−10^. Additionally, *A. thaliana* CESA, CSLA, CSLB, CSLC, CSLD, CSLE and CSLG were used as queries to identify homologous proteins in soybean with a blast score greater than 500. Only non-redundant sequences were kept and the “Cellulose_synt” domains in soybean CS proteins were confirmed by the SMART9.0 online tool (http://smart.embl-heidelberg.de/ accessed on 21 May 2024).

The theoretical isoelectric point and molecular weight of identified proteins were predicted by ExPASy (https://prosite.expasy.org/ accessed on 28 May 2024). Transmembrane domain was analyzed by TMHMM 2.0 (https://services.healthtech.dtu.dk/service.php?TMHMM-2.0 accessed on 28 May 2024). Protein subcellular locations were predicted using Cell-PLoc 2.0 database (http://www.csbio.sjtu.edu.cn/bioinf/Cell-PLoc-2/ accessed on 4 June 2024).

Multiple sequence alignment of CS proteins was performed using ClustalW in MEGA 7.0 with default parameters. A phylogenetic tree was constructed using Neighbor-Joining method based on the *P*-distance model with 1000 bootstrap replicates in MEGA 7.0. The phylogenetic tree was visualized and annotated by iTOL V7 online tool (https://itol.embl.de/ accessed on 10 June 2025).

### 4.2. Gene Structure Analysis and Conserved Domain Detection of CS

The coding sequence and genomic sequences of the *CS* genes were downloaded from Phytozome V14. Then gene structures (exon, intron, upstream and downstream) were subsequently visualized using the GSDS2.0 online software (http://gsds.cbi.pku.edu.cn/ accessed on 14 June 2024).

To identify conserved motifs, the CS protein sequences were submitted to the MEME V5.5.7 online suite (http://meme-suite.org/tools/meme accessed on 19 June 2024). The parameters were set as follows: motif width range of 6–50 amino acids and a maximum of 10 motifs.

### 4.3. Collinearity Analysis and Ka/Ks Calculation of CS Genes

Genome sequences and annotation files for multiple species were obtained from EnsemblPlants (https://plants.ensembl.org/index.html accessed on 1 September 2025), including *Glycine max* (Glycine_max_v2.1), *Arachis hypogaea* (arahy.Tifrunner.gnm2.J5K5), *Solanum lycopersicum* (SL3.0), *Arabidopsis thaliana* (TAIR10), *Triticum aestivum* (IWGSC), *Oryza sativa* (IRGSP-1.0), and *Zea mays* (Zm-B73-REFERENCE-NAM-5.0). Collinearity analysis of *CS* genes within the soybean genome and across species was performed using the One Step MCScanX method. These results were visualized using the Advanced Circos and Dual Systeny Plot for MCscanX module in TBtools V2.362 [64], respectively.

To evaluate selection pressure on duplicated *CS* genes in soybean, we calculated the non-synonymous (Ka) values, synonymous (Ks) values, and Ka/Ks ratios for each gene pair using “Simple Ka/Ks Calculator” module in TBtools V2.362. The selection status was classified as positive selection, purifying selection, or neutral selection based on the calculated Ka/Ks ratio being greater than, less than, or equal to 1, respectively [65].

### 4.4. Expression Analysis of CS Genes in Different Soybean Tissues

Tissue expression data (Fragments Per Kilobase of exon model per Million mapped fragments, FPKM) for all soybean *CS* genes across eight different tissues (pod, leaf, root, nodule, seed, shoot apical meristem (SAM), stem, flower) was downloaded from Phytozome V14 database. The FPKM values were subsequently transformed using the following formula:

Expression level = Log_2_ (FPKM + 1)

### 4.5. Promoter Cis-Acting Element Analysis

The 2000 bp promoter sequences upstream from the transcription start site of *CS* genes were submitted to the PlantCARE database (http://bioinformatics.psb.ugent.be/webtools/plantcare/html/ accessed on 4 September 2025) for *cis*-acting element analysis. The identified *cis*-elements were categorized into groups related to abiotic and biotic stress, phytohormone response, plant growth and development. The abundance of each *cis*-acting element type was quantified and visualized in a heatmap.

### 4.6. Plasmid Construction and Silencing of GmCESA1 in Soybean Plants

A VIGS system based on BPMV was kindly provided by John H. Hill (Iowa State University) [66], consists of two plasmids: the helper plasmid BPMV-R1M and the gene insertion plasmid BPMV-V2. A 360 bp fragment of the *GmCESA1* coding sequence was amplified from Williams 82 cultivar using primers (Supplementary Table S3) specific to *GmCESA1*, and cloned into the BPMV-V2 vector to generate the BPMV-V2-*GmCESA1* recombinant plasmid using ClonExpress MultiS One Step Cloning Kit (C113-01, Vazyme, Nanjing, China). Soybean leaves of Williams 82 were co-infected with equimolar mixture of the BPMV-V2-*GmCESA1* (or empty BPMV-V2) and BPMV-R1M plasmid. Three weeks post-inoculation, we generated *GmCESA1*-silenced (VIGS-*GmCESA1*) and empty vector control plants (VIGS-EV).

### 4.7. RNA Extraction and Gene Expression Analysis

Total RNA was extracted from plant tissues using the Plant RNA Extract Kit (DP432, TIANGEN Biotech, Beijing, China). cDNA was synthesized using a commercial reverse transcription kit (AG11728, Accurate Biology, Changsha, China). Reverse transcription quantitative polymerase chain reaction (RT-qPCR) was performed using SYBR Green Premix Pro Taq HS qPCR Kit (AG11701, Accurate Biology, Changsha, China) on LightCycler480 System (Roche Diagnostics, Rotkreuz, Switzerland). Gene-specific primers for *GmCESA1* were designed by Primer Premier V5 and listed in Supplementary Table S3, with *Gm60S* as the reference gene for normalization. The relative expression level of *GmCESA1* was calculated by 2^−ΔΔCt^ method. Three independent biological replicates were analyzed for each sample. All procedures were performed according to the manufacturers’ protocols.

### 4.8. Drought Treatment of Soybean and Determination of Physiological Indices

VIGS-*GmCESA1* and VIGS-EV soybean plants were grown to V3 stage (14 days after germination) in greenhouse. The plants were then subjected to either normal irrigation or simulated DS by watering with 20% PEG6000 solution for 21 days [67]. The growth conditions were maintained at 28 °C/24 °C (day/night) with a 14 h/10 h photoperiod. All phenotypic evaluations followed a randomized complete block design.

Relative water content (RWC) was determined following a previously published method [68] with minor modifications. Briefly, the third trifoliolate leaves were collected and fresh weight (FW) was immediately recorded. The leaves were then soaked in distilled water for 24 h to reach full turgor and obtain the saturated fresh weight (SFW). Finally, the leaves were dried in an oven at 80 °C for 24 h to measure the dry weight (DW). RWC was calculated as follows:

RWC (%) = [(FW − DW)/(SFW − DW)] × 100

Relative electrical conductivity (REC) was measured according to a previous protocol [69]. Briefly, 0.1 g of fresh third trifoliolate leaves was chopped and immersed in deionized water for 24 h at room temperature. The initial electrical conductivity (EC1) of the solution was measured using a conductivity meter (DDBJ-350, Lei Ci, China). The samples were then boiled for 20 min, cooled to room temperature, and the final electrical conductivity (EC2) was measured. REC was calculated as follows:

REC (%) = [EC1/EC2] × 100.

### 4.9. Measurement of Water Loss

For *A. thaliana*, entire rosette leaves were collected from 21-day-old plants grown under normal conditions, and the initial weight (W_0_) was immediately recorded. The rosette leaves were then placed in a dark environment with constant temperature and humidity. Their weight was measured hourly and recorded as W_t_ over a 6-h period [39].

For soybean, the middle leaflets of the third trifoliolate leaves above the cotyledonary node were excised from 5-week-old plants grown under normal conditions, and W_0_ was recorded. Leaf weight was measured every 6 h and recorded as W_t_ over a 24-h period. The water loss was calculated as follows:

Water loss (% of initial fresh weight) = [(W_0_ − W_t_)/W_0_] × 100

### 4.10. Drought Treatment of Arabidopsis thaliana

A mannitol-simulated drought stress assay was performed on *A. thaliana* based on previously described methods [39] with modifications. Seeds of the Columbian ecotype (Col) and *cesa1^rsw1-1^* [70] were surface-sterilized and vernalized in sterile water at 4 °C for 4 days and then sown on half-strength Murashige Skoog (1/2 MS) solid medium. After 5 days of growth in culture dishes at 22 °C under 16 h/8 h (day/night) photoperiod, uniformly sized seedlings were transferred to either control condition (1/2 MS solid medium containing 0 mM mannitol) or drought-simulating condition (1/2 MS solid medium containing 200 mM mannitol). Root length was measured at day 0 and day 5 after transfer, and root growth is the difference between the root length at day 5 and day 0. The relative root growth (RRG) was calculated as follows:

RRG = root growth__200 mM mannitol_/root growth __0 mM mannitol_

### 4.11. Measurement of Arabidopsis thaliana Rosette Area and Biomass

Seeds of Col and *cesa1^rsw1-1^* were sown in a growth medium consisting of vermiculite to nutrient soil at a 2:1 ratio (*v*/*v*). After seven days, seedlings were thinned to two plants per pot. The total rosette area of *A. thaliana* was measured non-destructively at 10, 15, 18, 21, 24, 27, and 30 days after sowing using a PhenoMate High-throughput Small Plant Phenotyping System (PhenoVation, Enschede, The Netherlands). Rosette dry weight was measured at 30 days after sowing.

### 4.12. Measurement of Cellulose Content

Rosette leaves from 30-day-old *A. thaliana* plants were collected for cellulose content measurement using commercial kits (BC4285, Solarbio, Beijing, China), following the manufacturer’s instructions.

### 4.13. Data Analysis

Data analysis was performed using Microsoft Excel 2019. Significant differences between two samples were determined using two-tailed Student’s *t*-tests. A *p*-value of less than 0.05 was considered statistically significant. Bar charts and line graphs were drawn using GraphPad Prism version 8.0.2.

## 5. Conclusions

This study identified 80 members of the cellulose synthase (CS) superfamily in soybean and provides a comprehensive bioinformatics characterization of soybean *CS* genes. Our results demonstrate a pivotal role of *GmCESA1* in drought tolerance and plant growth. Functional investigation using VIGS showed that silencing *GmCESA1* compromises drought tolerance and leads to reduced plant height in soybean. Further characterization of its homolog, *AtCESA1*, in *A. thaliana* confirmed the conserved function of *CESA1* in drought tolerance and plant growth. Together, these findings suggest *GmCESA1* is a pleiotropic and promising candidate gene for breeding soybean varieties with improved drought tolerance and growth traits.

## Data Availability

The original contributions presented in this study are included in the article/Supplementary Materials. Further inquiries can be directed to the corresponding author.

## Supplementary Materials

The following supporting information can be downloaded at: https://www.mdpi.com/article/10.3390/plants15010034/s1. Figure S1: Structural features of soybean *CS* genes; Figure S2: Conserved motif compositions of CS proteins in soybean; Figure S3: Heatmap of *CS* gene expression profiles across different soybean tissues; Table S1: The 80 members of CS superfamily identified in soybean; Table S2: Ka and Ks values of the *CS* gene pairs; Table S3: Primers used in this study.

## Abbreviations

The following abbreviations are used in this manuscript:

BPMV	Bean pod mottle virus
CS	Cellulose synthase
CSL	Cellulose synthase-like
DS	Drought-stress
REC	Relative electrical conductivity
RWC	Relative water content
NT	Nan tong xiao yuan dou
PI	PI595843
VIGS	Virus-induced gene silencing
