# Supplementary material for: Genome-Wide Analysis of Cellulose Synthase Superfamily and Roles of GmCESA1 in Regulating Drought Tolerance and Growth of Soybean
Source: Plants (Basel). 2025 Dec 22;15(1):34. doi: 10.3390/plants15010034 (PMC12787637; doi:10.3390/plants15010034)
Supplement: Supplementary file 1 [file plants-15-00034-s001.zip › plants-CESA1-Supp Figures-2025-12-7.pdf]

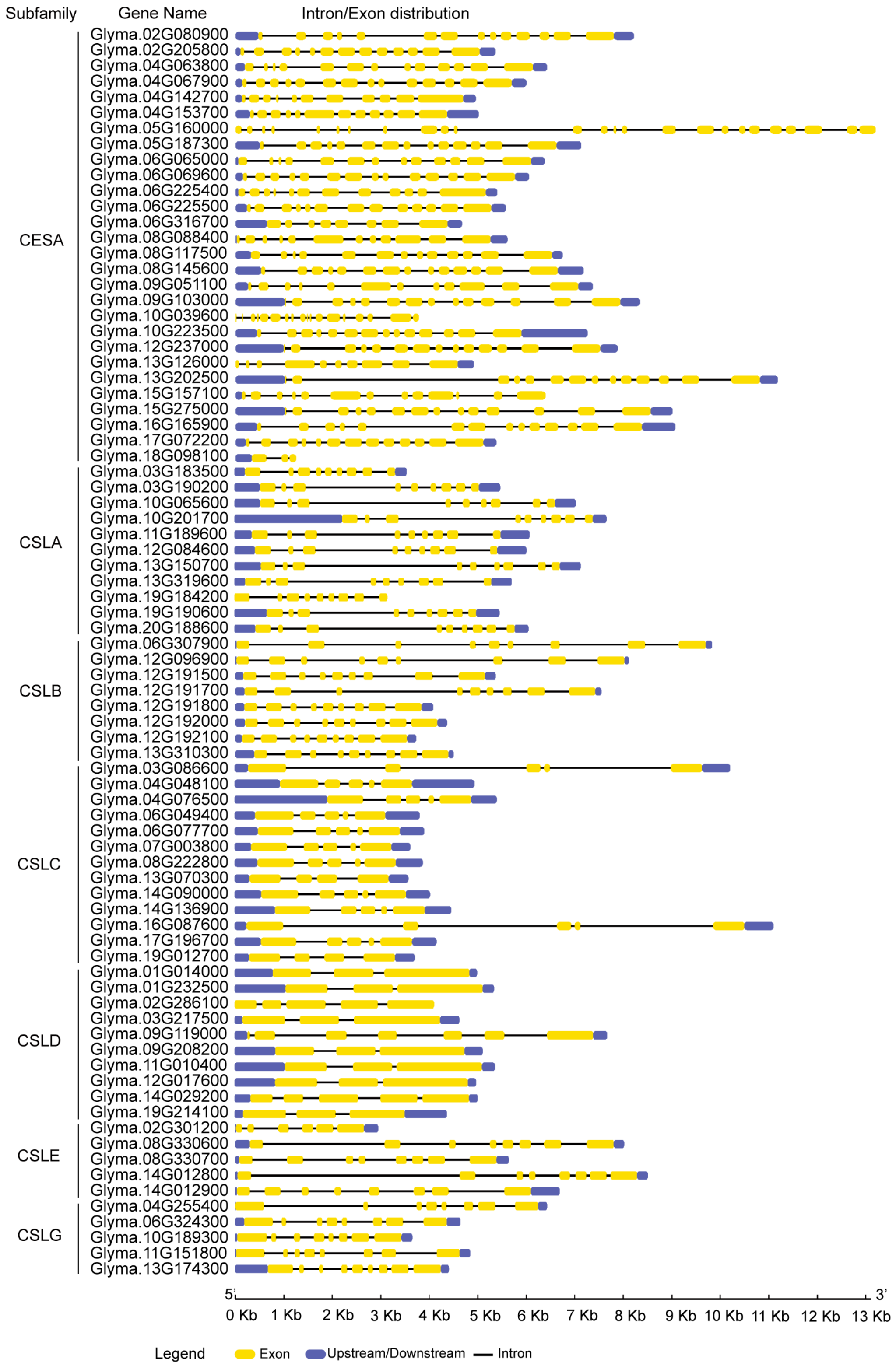

Supplementary Figure S1. Structural features of soybean *CS* genes. The exon-intron distributions and their relative positions are displayed, with a kilobase scale indicated at the bottom. Yellow boxes represent exons and black lines represent introns, and blue boxes denote the upstream and downstream regions.

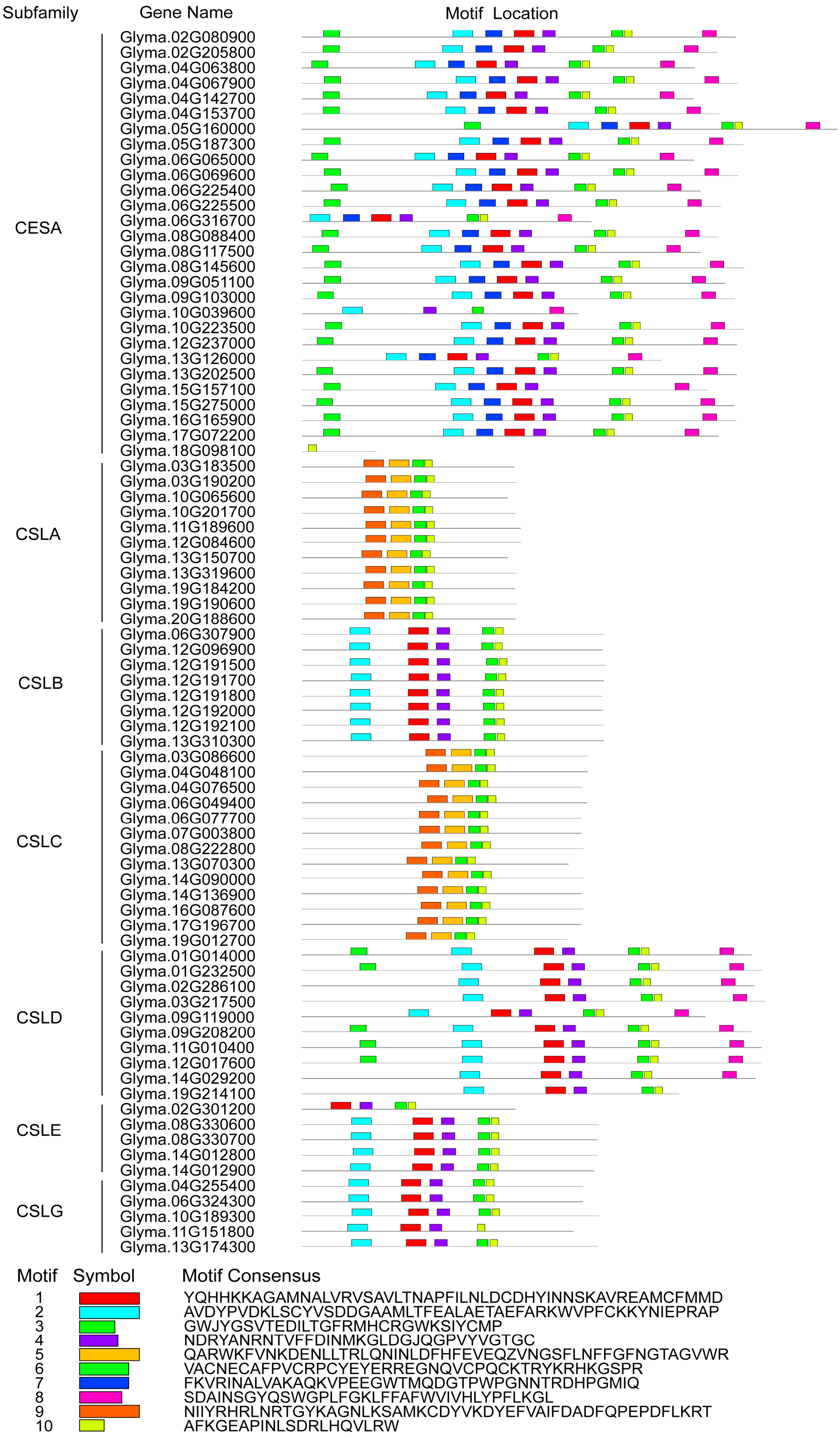

Supplementary Figure S2. Conserved motif compositions of CS proteins in soybean. The ten identified motifs are color-coded, and their details are provided at the bottom.

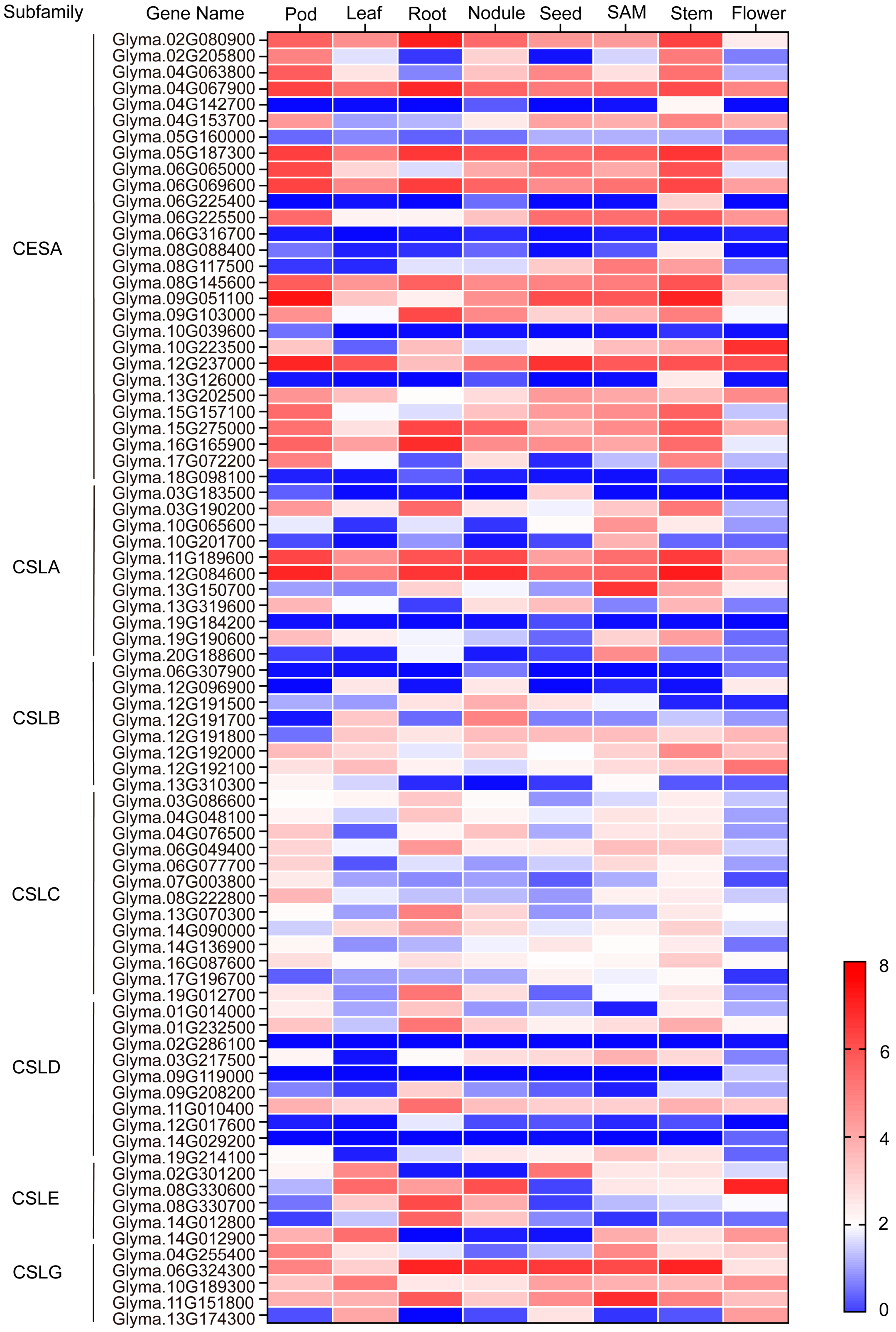

Supplementary Figure S3. Heatmap of *CS* gene expression profiles across different soybean tissues. The expression values (FPKM) were obtained from Phytozome V14 database and transformed as Log_2 (FPKM + 1) for visualization, with the color scale indicating relative transcript abundance.
